# Occurrence and Patterns of Enterotoxin Genes, *spa* Types and Antimicrobial Resistance Patterns in *Staphylococcus aureus* in Food and Food Contact Surfaces in Singapore

**DOI:** 10.3390/microorganisms11071785

**Published:** 2023-07-10

**Authors:** Ker Li Lim, Wei Ching Khor, Kar Hui Ong, Lois Timothy, Kyaw Thu Aung

**Affiliations:** 1 National Centre for Food Science, Singapore Food Agency, 7 International Business Park, Techquest, Singapore 609919, Singapore; lim_ker_li@sfa.gov.sg (K.L.L.); khor_wei_ching@sfa.gov.sg (W.C.K.); karhuiong7@gmail.com (K.H.O.); 2 School of Biological Sciences, Nanyang Technological University, 60 Nanyang Dr, Singapore 637551, Singapore; loistimothy19@gmail.com

**Keywords:** *Staphylococcus aureus*, *spa* type, antimicrobial resistance, retail food, food contact surface, enterotoxin genes

## Abstract

*Staphylococcus aureus* contamination of food and food contact surfaces is a public health concern given its virulent and antimicrobial-resistant properties worldwide. In this study, a total of 181 MSSA isolates were analyzed for SE genes, antimicrobial resistance patterns, and *spa* types. Overall, 24.9% of isolates were positive for SE gene detection, with *sea* being the most prevalent classical SE (18.8%). The most predominant sample sources for SE gene contamination were hand swabs for *sea* (6/48), meat dishes for *seb* (3/14) and seafood dishes for *sec* (2/24). Antimicrobial resistance was also observed at relatively high frequencies for the clinically important antibiotics penicillin G and ampicillin (both 54.7%), followed by tetracycline (14.9%) and azithromycin (8.8%). In addition, characterization of *spa* types revealed *spa* type t5078 to be the most predominant (40.3%), with significant associations between *spa* types t127 and t5521 and the *sea* gene. This study offers insights into the enterotoxin gene and antimicrobial resistance profiles of *S. aureus* in cooked or ready-to-eat food to inform future surveillance and epidemiological studies.

## 1. Introduction

*Staphylococcus aureus* has been recognized as a ubiquitous pathogen responsible for Staphylococcal food poisoning (SFP), a gastrointestinal intoxication resulting from the ingestion of food contaminated by enterotoxigenic *S. aureus* [1]. While *S. aureus* does not form spores, their opportunistic nature encourages growth in a wide range of temperatures (7 to 48.5 °C) and pHs (4.2 to 9.3) [2]. These characteristics promote the growth and spread of *S. aureus* in many food products, especially meat and meat products, poultry and egg products, unpasteurized milk, and dairy products [3]. After contamination, improper storage conditions and poor hygiene practices accelerate the growth of *S. aureus*, allowing it to reach the cell density necessary for enterotoxin production. Hence, it is crucial that proper hygiene standards are adhered to during food processing and storage to minimize the spread and growth of the pathogen and its enterotoxins throughout the food processing chain.

Staphylococcal enterotoxins (SEs) produced by coagulase-positive *staphylococci* are the main causatives agents of SFP. SEs are resistant to heat, proteolytic enzymes, and other environmental conditions [4]. Due to their stable nature, the detection of SEs is a definite method for the confirmation of outbreaks and the enterotoxigenicity of strains. There are over 20 *S. aureus* enterotoxins identified. Based on serological classification, they are grouped as classical genes and non-classical genes (new SEs). Classical genes are the top five predominant enterotoxins (*sea*, *seb*, *sec*, *sed*, and *see*) that are highly isolated from outbreaks of SFP in more than 90% of cases, while non-classical genes are new enterotoxins that were isolated from 5% of cases [5]. Due to the stable properties of SEs and the low dose required to cause symptoms, consumption of food contaminated with enterotoxigenic *S. aureus* can easily lead to foodborne outbreaks. There are several reported SFP outbreaks across Asia [6,7,8], though none were reported in Singapore. Nonetheless, contamination of *S. aureus* with food and food contact surfaces is a public health concern given its virulent and antimicrobial-resistant properties worldwide.

Molecular typing is a useful tool to understand clonal relatedness, genetic diversity, and the spread of pathogens [9]. There are several molecular methods to identify enterotoxigenic *S. aureus* strains, including pulsed-field gel electrophoresis (PFGE), multi-locus sequence typing (MLST), whole genome sequencing (WGS), and *spa* typing. In this study, *spa* typing was adopted. *Spa* typing is a single-locus typing method to analyze the highly variable X region of the protein A gene in *S. aureus* strains. The X region consists of 24-bp tandem repeats flanked by well-conserved regions. Repeats are assigned a unique repeat code, and the *spa* type of a given strain is derived from the number of tandem repeats and the sequence variation in the X region. Due to its discriminatory power to identify strains based on a single-locus DNA sequence-based marker in the presence of polymorphisms, this method proved to be as effective as other typing methods, including PFGE and MLST [10]. It is also most cost-effective, less time-consuming, and less error-prone compared to other molecular methods [9,11]. While WGS can differentiate closely related strains with greater sensitivity as compared to *spa* typing, the *spa* gene is composed of highly variable and similar repeats, which could pose a challenge for WGS since repeated sequences can be misassembled [12]. Since the objective of our study is to understand if there is a possible transfer of *S. aureus* from food handlers to food, the use of *spa* typing in our study would be sufficient and more cost-efficient compared to WGS, so *spa* typing is adopted in this study.

Over the past decades, the increasing use of antibiotics in animal and human medicine has led to an increasing public health concern about antibiotic resistance in pathogens, including *S. aureus*, [4]. *S. aureus* also colonizes animals, and transmission between humans and animals has been reported [1]. Once humans acquire it, community transmission is possible. With the increasing use of antibiotics in animals, the emergence of antimicrobial resistance and increasing virulence would be a public health concern [1]. Although SFP is mostly self-limiting in healthy adults, treatment with antimicrobials is necessary for invasive and immunocompromised individuals [13], and the presence of resistance traits can render corresponding antimicrobials ineffective in treating the infection or intoxication, resulting in serious public health issues [13].

To the best of our knowledge, limited research has been done in Singapore on *S. aureus* in the food chain and its implications for food safety with regards to antimicrobial resistance and enterotoxigenicity. Additionally, SFP is not a notifiable disease in Singapore. Therefore, the data on the incidence of SFP in the population is limited. Hence, the objectives of this study are to evaluate the occurrence and prevalence of *S. aureus* strains in food and food contact surfaces in Singapore and to examine the antimicrobial susceptibility pattern of these strains. Through an understanding of the molecular epidemiology of these strains in retail food and food handlers, findings from this study will be useful to inform public health and mitigation measures at the retail level, such as good food handling practices among food handlers, and strengthen future surveillance and epidemiological studies.

## 2. Materials and Methods

### 2.1. Sample Collection, Isolation and Identification of S. aureus

A total of 1540 retail food and food contact surface samples were obtained from food surveillance and risk assessment studies conducted by the National Environmental Agency between 2009 and 2013.

A ten-gram sample of each food item was placed in a sterile stomacher bag and homogenized with 90 g of Universal Pre-enrichment Broth (UPB) (Acumedia Manufacturers, Lansing, MI, USA) using a stomacher (Seward Stomacher^®^ 400 Circulator, Seward, West Sussex, UK) at 230 rpm for 30 s. Serial 10-fold dilutions were prepared using 9 mL of Butterfield’s buffer (3M, St. Paul, MN, USA). For the detection of *S. aureus*, 1 mL of a 10-fold diluted sample was equally distributed between two plates of Baird-Parker agar (Oxoid, Basingstoke, Hants, UK) before incubation at 37 °C for 48 h. Presumptive *S. aureus* colonies (grey-black colonies with a narrow white margin surrounded by a zone of clearing) were tested for a catalase reaction using 3% hydrogen peroxide (ICM Pharma, Singapore) and confirmed using coagulase rabbit plasma (Remel, Haverhill, MA, USA).

### 2.2. Detection and Isolation of SE Genes

The detection of SE genes was performed using the following method [14,15,16,17].

DNA was extracted from pure *S. aureus* colonies grown on Tryptic Soy Agar plates with 5% sheep blood (Acumedia, Baltimore, MD, USA) using the QIAamp^®^ DNA Mini Kit (Qiagen, Hidden, Germany). Multiplex and singleplex PCR assays were performed to detect virulence genes (*sea*, *seb*, *sec*, *sed*, *see*, *seg*, *seh*, *sei*, *sej*, and *sel*) and the *mecA* gene characteristic of methicillin-resistant *S. aureus* (MRSA).

PCR master mixes were prepared as shown below (Table 1). Each PCR mix (45 μL) consists of 5× Phusion High-Fidelity Buffer (Thermo Scientific, Vilnius, Lithuania), dNTP mix (1st BASE, Seri Kembangan, Malaysia), 10 μM of each primer (Integrated DNA Technologies, Singapore) (Table 2), Phusion Hot Start II DNA Polymerase (Thermo Scientific, Vilnius, Lithuania), DNA template, and molecular-grade water. *S. aureus* 29213, *S. aureus* 43300, *S. aureus* ATCC^®^ 13565, *S. aureus* ATCC^®^ 14458, *S. aureus* ATCC^®^ 23235, *S. aureus* ATCC^®^ 27664, *S. aureus* ATCC^®^ 19095, and BAA *S. aureus* ATCC^®^ 1761 were used as positive controls, while molecular-grade water was used as a negative control.

Amplification using multiplex PCR was conducted using the following parameters: initial denaturation of the strand at 98 °C for 30 s, followed by 30 cycles of denaturation at 98 °C for 10; annealing at 61 °C for 30 s; extension at 72 °C for 30 s; and final extension for 10 min at 72 °C. For amplification using singleplex PCR, the following parameters were used: initial denaturation of the strand at 98 °C for 30 s, followed by 35 cycles of denaturation at 98 °C for 10 s; annealing at 57 °C for 30 s; extension at 72 °C for 30 s; and final extension for 10 min at 72 °C. PCR-positive MSSA samples were confirmed with a latex agglutination test (PBP2) (Oxoid) and a cefoxitin disc (Oxoid) using the disc diffusion method following Clinical and Laboratory Standards Institute (CLSI) guidelines [18,19].

The amplified products were visualized using gel electrophoresis on a 1.5% agarose gel for multiplex PCR 1, singleplex PCR 1 and 2, and a 2% agarose gel for multiplex PCR 2. Detectable PCR bands were confirmed to contain the virulence genes.

### 2.3. Spa Typing

The *spa* typing for the *S. aureus* isolates was performed using the following method [20,21,22,23].

The X region of the protein A gene was amplified using PCR with four primer sets: 1113f and 1514r; 1095f and 1517r; 1084f and 1618r; and 238f and 1717r. If no PCR amplification was detected with one of the primer sets, the other three sets were used for PCR amplification instead.

The PCR mix for *spa* typing consists of 10 µL of 5× Phusion High-Fidelity Buffer (Thermo Scientific, Vilnius, Lithuania), 1 µL of dNTP mix (10 mM) (1st BASE, Seri Kembangan, Malaysia), 0.5 µL of each forward and reverse primer (Integrated DNA Technologies, Singapore), 0.5 µL of Phusion Hot Start II DNA Polymerase (Thermo Scientific, Vilnius, Lithuania), and 32.5 µL of molecular-grade water. To each PCR mix, 5 µL of DNA template was added.

Amplification was conducted using the following parameters: For primers 1113f and 1514r, initial denaturation of the strand at 98 °C for 30 s is followed by 35 cycles of denaturation at 98 °C for 10 s, annealing at 61 °C for 30 s, extension at 72 °C for 30 s, and final extension for 10 min at 72 °C. For primers 1095f and 1517r, initial denaturation of the strand at 98 °C for 30 s is followed by 35 cycles of denaturation at 98 °C for 10 s, annealing at 45 °C for 30 s, extension at 72 °C for 30 s, and final extension for 10 min at 72 °C. For primers 1084f and 1618r, 238f and 1717r, initial denaturation of the strand at 98 °C for 30 s is followed by 35 cycles of denaturation at 98 °C for 10 s, annealing at 55 °C for 30 s, extension at 72 °C for 30 s, and final extension for 10 min at 72 °C. The amplified products were visualized using gel electrophoresis on 1.5% agarose gel. Detectable PCR bands were confirmed to contain the *spa* gene.

PCR products were purified using the QIAquick^®^ PCR Purification Kit (Qiagen, Hilden, Germany) and sequenced by capillary electrophoresis using BigDye Terminator chemistry (AIT Biotech, Singapore). Sequences were analyzed using BioNumerics v7.6 to determine *spa* types.

### 2.4. Antimicrobial Susceptibility Testing

Antimicrobial susceptibility for *S. aureus* isolates was determined by the disc diffusion method according to the Clinical and Laboratory Standards Institute (CLSI) guideline [18]. All the antibiotics used to determine antimicrobial resistance were grouped into nine classes: Aminoglycosides, Beta-lactams, Cephalosporins, Chloramphenicols, Fluoroquinolones, Glycopeptides, Macrolides, Sulphonamides and Tetracyclines. The antimicrobial agents used were Ciprofloxacin (CIP5), Norfloxacin (NOR10), Amikacin (AK30), Ampicillin (AMP10), Gentamicin (CN10), Tetracycline (TE30), Ceftriaxone (CRO30), Amoxycillin/Clavulanic acid (AMC30), Sulphamethoxazole/Trimethoprim (SXT25), Chloramphenicol (C30), Azithromycin (AZM15), Penicillin G (P10), Vancomycin (VA30), Cefoxitin (FOX30), and Rifampicin (RD5). The zone diameter breakpoints used were obtained from the CLSI standards [18]. *Staphylococcus aureus* ATCC^®^ 25923 was used as the quality control strain, while sterile water was used as a negative control.

### 2.5. Statistical Analysis

All statistical analyses were performed using GraphPad Prism 8.0 (GraphPad Software, LLC, San Diego, CA, USA). A *p*-value < 0.05 was considered statistically significant. Non-random associations between categorical variables (*spa* type and SE gene) were determined using the Fisher’s exact test. Cluster analysis was performed on BioNumerics v7.6 and a maximum distance of 2 was used to determine closely related *spa* types in the same cluster.

The 95% confidence intervals of proportions were calculated using http://vassarstats.net/prop1.html (accessed on 23 March 2023) Z-scores for two population proportions were calculated using https://www.socscistatistics.com/tests/ztest/default2.aspx (accessed on 1 April 2023).

## 3. Results

### 3.1. Occurrences and Distribution of SE Genes

The prevalence of *S. aureus* in food and food contact surfaces is 15.4% (237/1540 samples). All 237 *S. aureus* isolates were determined to be methicillin-susceptible *S. aureus* (MSSA). Of the 237 *S. aureus* isolates tested, 181 could be typed using *spa* sequencing. The remaining strains that could not be typed were excluded from the analysis. The frequency of isolates from food and food contact surfaces are shown in Table 3.

Of the 181 isolates, 45 (24.9%) [95% CI: 19.1–31.6%] were detected with at least one of the SE genes. A total of 96 SE genes were detected among these 45 isolates.

The occurrence of each SE gene across all isolates is shown in Table 4 below.

The most common classical SE gene is the *sea* gene, while the most common non-classical genes are *seg* and *sei*. In total, *seg* and *sei* genes had the highest occurrence (25/96) (26.0%) across all SE genes. *sed* and *see* genes were not detected in any of the isolates tested in this study.

**Figure 1:**
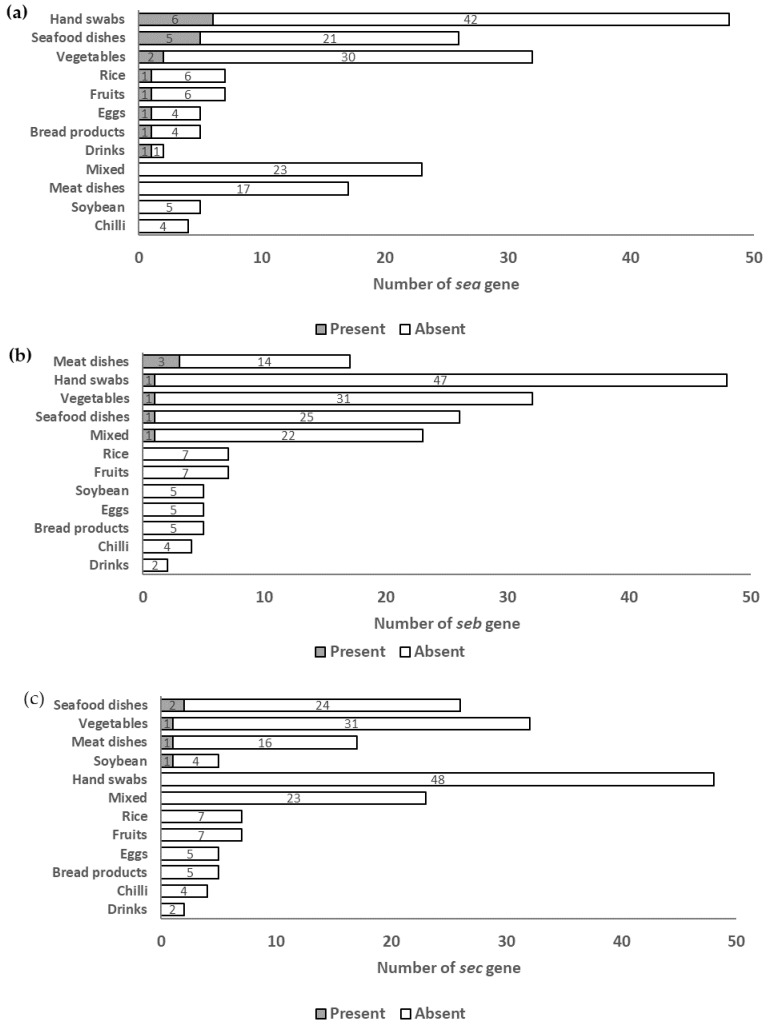
Distribution of (**a**) the *sea* gene; (**b**) the *seb* gene; and (**c**) the *sec* gene across categories of food and food contact surface.

The predominance of classical SE genes was categorized based on their food and food contact surface categories, as shown in Figure 1 below. The *sea*, *seb* and *sec* genes were predominantly found in hand swabs (6/48), meat dishes (3/17) and seafood dishes (2/26) respectively.

### 3.2. Occurrence and Distribution of Antimicrobial Resistance (AMR) in Food

The percentage of antimicrobial resistance in *S. aureus* isolates are shown in Table 5. All 181 *S. aureus* isolates were susceptible to ceftriaxone, cefoxitin, chloramphenicol, rifampicin, sulfamethoxazole/trimethoprim, and vancomycin. The occurrence of resistance was 54.7% against ampicillin (99/181), 54.7% against penicillin G (99/181), 14.9% against tetracycline (27/181), and 8.8% against azithromycin (16/181).

Table 6 shows the classification of antimicrobial agents in different food and food contact surface categories. Noticeably, isolates that were resistant to ampicillin were resistant to penicillin G across all categories. In addition, resistance to tetracycline and azithromycin was observed in most of the categories.

### 3.3. Distribution of Spa Types

**Figure 2:**
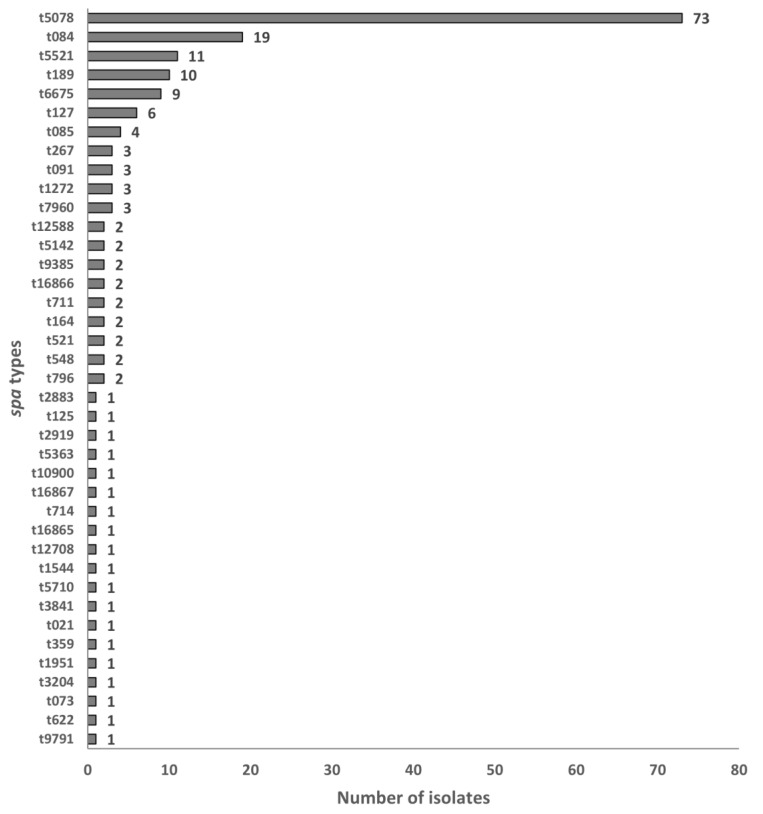
Distribution of *spa* types across all isolates.

Figure 2 shows the distribution of *spa* types for the *S. aureus* isolates. All 181 *S. aureus* isolates were classified under 39 *spa* types. The top six predominant *spa* types were t5078 (73/181, 40.3%), t084 (19/181, 10.5%), t5521 (11/181, 6.1%), t189 (10/181, 5.5%), t6675 (9/181, 5.0%), and t127 (6/181, 3.3%). These *spa* types were analyzed for the presence of SE genes, as shown in Table 7.

The most predominant classical SE gene, *sea*, was observed to have the highest proportion in t5521, representing 73% (11/15) of all *sea*-positive isolates. The associations between *spa* type t5521 and the presence of the *sea* gene (*p* < 0.0001) and between *spa* type t127 and the *sea* gene (*p* = 0.0138) were determined to be statistically significant using Fisher’s exact test.

**Figure 3:**
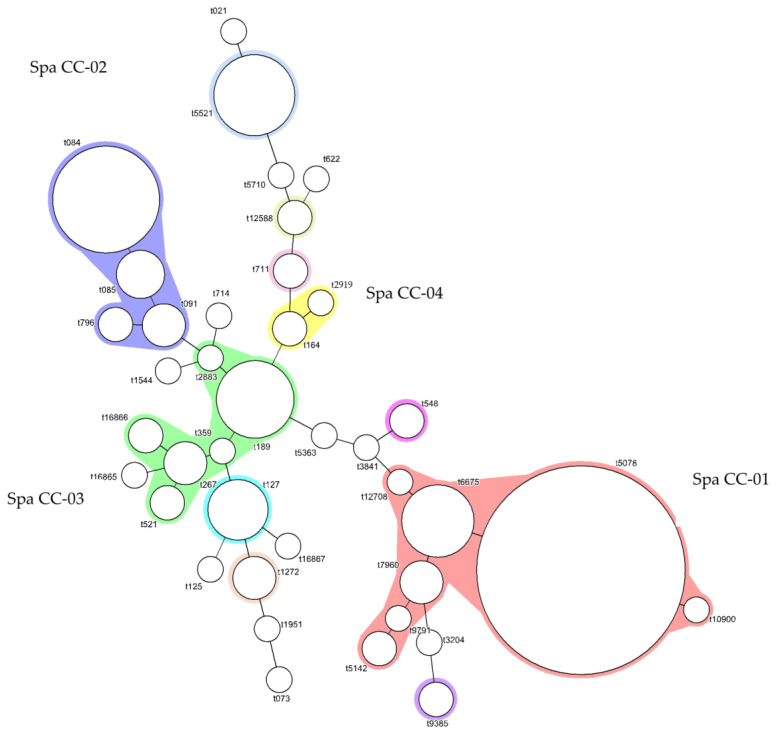
Minimum-spanning tree of spa types from the 181 isolates.

A minimum-spanning tree was constructed to perform *spa* clustering analysis for all isolates (Figure 3). Clusters were arbitrarily assigned to clustering complexes *spa* CC01 to *spa* CC04, according to the four definitive clusters observed. *Spa* types were partitioned into complexes when the distance between connected nodes was ≤2.

## 4. Discussion

### 4.1. Overall Occurrence of SE Genes

In this present study, 24.9% (45/181) of the isolates were detected for the presence of at least one SE gene. The incidence of *S. aureus* detected with at least one or more SE genes in this present study (24.9%) was relatively lower than that reported in other countries, such as Korea (48.0%), China (54.4%), and Italy (55.5%) [24,25,26], which was expected. One of the most common foods associated with SFP is milk and dairy products [3]. In Singapore, only heat-treated milk is permitted to be sold for direct human consumption [27], hence there is a lower risk of *S. aureus* contamination as compared to raw milk, which could be a possible reason for the lower incidence compared to other countries. Another possible reason is that food handlers in Singapore are required to undergo a compulsory food safety course to equip them with the basic hygiene knowledge required for handling food, resulting in a lower incidence of *S. aureus*. The lower incidence of *S. aureus* with SE genes also correlates with the occurrence of SFP outbreaks, as Singapore has no known reported SFP outbreaks compared to other Asian countries [6,7,8].

Among the ten SE genes tested, the classical SE gene *sea* (18.8%, 18/96) and the non-classical SE genes *sej* (26.0%, 25/96) and *sei* (26.0%, 25/96) were detected at the highest frequencies. For the classical SE gene *sea*, observation was similar to studies conducted in other countries, such as Taiwan (29.9%) and Iran (25.5%), where the *sea* gene was most prevalent among the classical genes [5,28]. The *sea* gene was most commonly isolated in cases of SFP and was frequently isolated in SFP outbreaks in Japan and the United States [3,8]. Enterotoxins *sea* and *seb* are known to cause approximately 90% of staphylococcal food poisoning worldwide [29]. The presence of enterotoxin genes in these isolates suggests the isolates’ potential to produce toxins under favorable conditions and cause staphylococcal food poisoning (SFP) if allowed to grow in large numbers in food.

In contrast, several other studies by Hait et al. [30] and Tang et al. [31] have found non-classical genes to be the most predominant genes detected in the isolates investigated. Non-classical genes are new types of genes that have lower expression than classical genes. A non-classical sei gene has been detected in food poisoning-associated *S. aureus* isolates in Switzerland, the United Kingdom, and Japan [32,33,34]. However, despite the presence of sei genes in these isolates, it remains an open question whether these isolates have produced enterotoxins in sufficient amounts in food to cause SFP. The detection of enterotoxin sei in food should be explored further to make an accurate association between the sei gene and its ability to cause food poisoning.

### 4.2. Occurrence of SE Genes according to Food and Food Contact Surface Category

Due to the widespread occurrence of classical genes among SFP outbreaks [5], this study will focus on the comparison of the occurrence of classical SE genes across food and food contact surfaces. Of the food and food contact surfaces, hand swabs had the highest incidence of the sea gene (12.5%, 6/48). This is similar to studies conducted in Brazil and Japan, where high occurrences of the sea gene were detected in hand swab samples [35,36]. Without proper hygiene practices, such as wearing gloves during food preparation, *S. aureus* can be transmitted from human skin to food. This suggests that food handlers without proper hygiene care may increase the risk of contamination in food, as they act as vectors for the spread of enterotoxigenic *S. aureus* to food [2,35,37], which increases the risk of consumers consuming food contaminated with enterotoxigenic *S. aureus*. Contamination by food handlers contributes significantly to food poisoning outbreaks. In the United States, 42% of outbreaks between 1975 and 1988 were attributed to contamination by food handlers [2].

Of the food and food contact surfaces, meat dishes had the highest incidence of enterotoxin *seb* (21.4%, 3/14). Previous studies have reported few or no detections of the *seb* gene in *S. aureus* isolates in retail meat samples [38,39], which is interesting to note as the *seb* gene is directly associated with human contamination [40]. Meat dishes collected in this study could be more susceptible to human contamination, as the dishes, including chicken rice and duck rice, involve post-cooking manipulation, such as cutting and shifting the meat from chopping board to plate. Potential contamination sources include cutting boards, knives, or improper hygiene practices by food handlers [24]. Similar to the *sea* gene, the *seb* gene has remarkable stability against heat and proteolytic digestion [29,41,42]. Contamination of food with the *seb* gene in suitable numbers could result in severe food poisoning as well [19]. The occurrence of the *sec* gene among *S. aureus* isolates was highest in seafood dishes (7.7%, 2/26). Similar findings were reported in a study where 12.5% (1/8) of fish products were contaminated with the *sec* gene [26].

The presence of enterotoxin genes in *S. aureus* isolates is not necessarily a definitive indication of protein expression in these genes, as these genes may be non-functional or silent due to point mutations [43]. In addition, the level of enterotoxin production is dependent on other factors, including pH, water activity, temperature, and other parameters [34]. Knowledge on the occurrence of enterotoxin genes in this study, therefore, does not reflect the true enterotoxigenic potential of the *S. aureus* isolates. This limitation calls for greater research into the expression of genes in isolates retrieved from food to inform exposure and quantitative microbiological risk assessment (QMRA). Nonetheless, the presence of *S. aureus* strains with multiple enterotoxin genes still presents a threat to public health with respect to the consumption of contaminated food and contamination by food handlers.

### 4.3. Distribution of Spa Types

Molecular characterization by *spa* typing revealed a wide genetic diversity with the identification of 39 *spa* types among all the food and food contact surface isolates, with *spa* type t5078 as the most prominent (40.3%, 73/181), followed by type t084 (10.5%, 19/181), t5521 (6.1%, 11/181), t189 (5.5%, 10/181), t6675 (5.0%, 9/181), and t127 (3.3%, 6/181). While there are several studies associating *spa* types with food and food contact surfaces [44,45,46], the *spa* types identified in this current study were more associated with patients and human blood isolates than food or food-related isolates.

*Spa* type t5078 has been linked to MSSA isolates isolated from patients in different countries. In Singapore, *spa* type t5078 was discovered in a MSSA isolate that was detected on an infected indwelling graft in a patient suffering from chronic renal failure [47]. In Taiwan, *spa* type t5078 was isolated from blood samples from patients, which were then discovered to be MSSA isolates [48]. According to Tunsjø et al. [49], *S. aureus* shares similar virulence genes, pathogenicity islands, and bacteriophages with *S. argenteus*. This is consistent with a study by Aung et al. [50], where 50% (12/24) of *S. argenteus* isolates were classified into *spa* type t5078 and other *spa* types with similar repeat profiles to t5078. *Spa* type t084 was found to be the most predominant *spa* type among MSSA isolates in a children’s hospital in Poland and in the United States, with reports of invasive infections and being present in healthcare-associated and community-onset infections [51,52]. In another study, *spa* type t084 was also one of the predominant *spa* types among MSSA isolates among healthcare workers and patients [53]. The third predominant *spa* type, t5521 (6.1%, 11/181), was not actively studied in many countries. In a study conducted by Uhlemann et al. [54], t5521 was identified as one of eight new *spa* types isolated from *S. aureus* isolates from patients in Martinique. However, as t5521 is a relatively new *spa* type, no further extensive research was conducted.

*Spa* type t127 was also associated with an MSSA outbreak caused by ice-cream in Germany, with a high concomitance with the *sea*, in concordance with the results of this study (*p* = 0.0138) [55]. This links *spa* type t127 to potential food poisoning events. However, the statistically significant association (*p* < 0.0001) between *spa* type t5521 and *sea* in this present study has not been reported in other studies to the best of our knowledge. Further research is recommended to validate the statistical associations, which can aid in surveillance and epidemiological analysis of *S. aureus* infections and SFP outbreaks [56].

One limitation of *spa* typing in this study was the high proportion of non-typable *spa* types (23.6%), either due to the low quality of tandem repeats or no sequence generated. Future studies could consider using WGS to evaluate the reliability of *spa* typing by PCR. *Spa*-typing has been effective in distinguishing *S. aureus* from various sources, which will be relevant and useful for the epidemiological determination of food sources in outbreak investigations. Although the *S. aureus* strains in this study were isolated from surveillance and risk assessment studies and not from outbreak investigations, studying the genetic patterns of *S. aureus* isolates in food and food contact surfaces will be useful to understand the molecular epidemiology of these isolates, which will be useful in cases of improper hygiene practices or food handling during food production and storage.

### 4.4. General Antimicrobial Resistance Patterns

In this study, resistance to beta-lactams, specifically penicillin G and ampicillin, was observed at the highest frequency (54.7%, 99/181). The results are in agreement with other reports regarding the resistance of *S. aureus* detected in food to penicillin G in the United States (67.4%), Kuwait (82.0%), China (83.7%), and Western Algeria (60.8%) [57,58,59,60]. Notably, ampicillin and penicillin G resistance occurred at the same frequency (54.7%), similar to the results observed in bovine milk samples in China (91.4%) [61] and MSSA isolates in Trinidad and Tobago (11%) [62]. Penicillin resistance through beta-lactamase is conferred by the *blaZ* gene, which can be chromosomal or plasmid-encoded [63,64]. Furthermore, the spread of Penicillin G resistance occurs with the spread of resistant strains of *S. aureus*, where food could act as a vector [63,65]. However, as many clinically relevant *S. aureus* strains do possess beta-lactamase functions [66], penicillin is unlikely to be used for treatment of SFP, and thus the high resistance to both penicillin G and ampicillin in most sample categories identified in this study could be inherent. However, this study showed limited resistance to amoxycillin/clavulanic acid (0.6%), perhaps owing to the beta-lactamase inhibition activity of clavulanic acid [67]. Therefore, it could be postulated that beta-lactams are resistant. *S. aureus* isolates in this study were likely due to the presence of beta-lactamase activity.

The findings from this current study also showed that tetracycline resistance was high (27/181, 14.9%). Other studies in the United States (56.4%) and China (24.4%) have reported varied resistance to tetracycline [57,68,69]. The varied resistance to tetracycline in different countries could be explained using the varying usage of tetracycline in animal feeds, and the treatment of bacterial infections in plants, agriculture, and human medicine [70]. While tetracycline resistance in this study is high, compared to other antimicrobial agents tested, the frequency is still considerably low compared to other countries and thus should not be a cause for concern.

To date, there is a limited understanding of the transmission of antimicrobial-resistant *S. aureus* through food and food-contact surfaces. Food provides a conducive environment for the growth of bacteria. In addition, food chains are important in the spread of antimicrobial resistance between food and the environment [71,72]. These suggest that ready-to-eat food and food contact surfaces can be potential environmental sources for the colonization and circulation of antimicrobial-resistant *S. aureus* in the community [37,71]. Antimicrobial resistant *S. aureus* will not be a food safety concern if enterotoxin genes are not expressed and allowed to grow in sufficient numbers in food. However, the consumption of food contaminated with enterotoxigenic *S. aureus* with antimicrobial resistance could pose a serious food safety and public health risk [59]. In addition, antimicrobial-resistant *S. aureus* in food could contribute to a larger part of the environmental resistome. Hence, it is crucial to monitor the antimicrobial resistance and enterotoxigenicity of MSSA in retail food to understand epidemiological changes and develop strategies to prevent the contamination of the pathogen in food.

### 4.5. Antimicrobial Resistance Patterns according to Food and Food Contact Surface Category

The results indicated that tetracycline resistant *S. aureus* was high in bread products (3/5, 60%). This was reported in China as well (23.3%) [59]. Studies conducted in other countries have shown the possibility of associating antimicrobial resistance with a particular type of food, such as in Iran, where chloramphenicol resistance was identified in food products made from poultry meat, which correlated to the use of chloramphenicol to treat infections in poultry [73]. Due to the limited availability of an equal number of isolates across different sample categories, this study did not have the chance to show that a particular food or food contact surface category was at increased risk of acting as a vehicle for antimicrobial transmission. More data and larger sample sizes are required to calculate risk ratios and draw conclusions about whether an association between antimicrobial resistance and food or contact surfaces is causal in nature.

## 5. Conclusions

In conclusion, this study analyzed the patterns of SE genes, *spa* types, and antimicrobial resistance of *S. aureus* in food and food contact surface samples. This study revealed the occurrence of antimicrobial-resistant or enterotoxigenic *S. aureus* in food and food contact surface samples, suggesting that food or food contact surfaces can be potential vehicles for spreading *S. aureus*. Hence, there is a need for constant monitoring of food hygiene. In addition, findings from this study offer epidemiological insights to inform future surveillance and quantitative microbiological risk assessment.

## Figures and Tables

**Table 1:** Volume of reagents used for multiplex and singleplex PCR assays in this study.

Reagents	Volume of Reagents Used for the Detection of Virulence Genes (µL)			
	Multiplex PCR 1	Multiplex PCR 2	Singleplex PCR 1	Singleplex PCR 2
HF Buffer (5×)	10	10	10	10
dNTP (10 mM)	1	1	1	1
ESA F + R primer (10 μM)	1			
ESB F + R primer (10 μM)	1			
ESC F + R primer (10 μM)			1	
ESD F + R primer (10 μM)	1			
ESE F + R primer (10 μM)	1			
ESG F + R primer (10 μM)		0.5		
ESH F + R primer (10 μM)		0.5		
ESI F + R primer (10 μM)		1		
ESJ F + R primer (10 μM)				1
ESL F + R primer (10 μM)		1		
Phusion Taq polymerase	1	0.5	0.5	0.5
Molecular grade water	29	30.5	32.5	32.5

**Table 2:** Nucleotide sequences and amplicon sizes for virulence gene primers used for this study.

Gene	Primer	Nucleotide Sequences	Amplicon Size (bp)	Multiplex/Singleplex PCR
*sea*	ESA′	5′-ACGATCAATTTTTACAG′-3′	544	Multiplex PCR 1
	ESA′	5′-TGCATGTTTTCAGAGTTAAT′-3′		
*seb*	ESB′	5′-GAATGATATTAATTCGCAT′-3′	416	Multiplex PCR 1
	ESB′	5′-TCTTTGTCGTAAGATAAACTT′-3′		
*sec*	ESC′	5′-GACATAAAAGCTAGGAATT′-3′	257	Singleplex PCR 2
	ESC′	5′-AAATCGGATTAACATTATCC′-3′		
*sed*	ESD′	5′-TTACTAGTTTGGTAATATCTCCT′-3′	334	Multiplex PCR 1
	ESD′	5′-CCACCATAACAATTAATG′-3′		
*see*	ESE′	5′-ATAGATAAAGTTAAAACAAGCA′-3′	170	Multiplex PCR 1
	ESE′	5′-TAACTTACCGTGGACC′-3′		
*seg*	ESG′	5′-ACGTCTCCACCTGTTGAAG′-3′	400	Multiplex PCR 2
	ESG′	5′-TGAGCCAGTGTCTTGCTTT′-3′		
*seh*	ESH′	5′-TCACATCATATGCGAAAGCA′-3′	357	Multiplex PCR 2
	ESH′	5′-TAGCACCAATCACCCTTTC′-3′		
*sei*	ESI′	5′-TGGAACAGGACAAGCTGAA′-3′	467	Multiplex PCR 2
	ESI′	5′-TAAAGTGGCCCCTCCATAC′-3′		
*sej*	ESJ′	5′-CAGCGATAGCAAAAATGAAAC′-3′	240	Singleplex PCR 1
	ESJ′	5′-TCTAGCGGAACAACAGTTCTG′-3′		
*sel*	ESL′	5′-CACCAGAATCACACCGCTT′-3′	426	Multiplex PCR 2
	ESL′	5′-CTGTTTGATGCTTGCCATT′-3′		

**Table 3:** Number and percentage of *S. aureus* isolates obtained for this study.

Sample Category	*n*	%
Hand swabs	48	26.5
Vegetables	32	17.7
Seafood dishes	26	14.4
Mixed	23	12.7
Meat dishes	17	9.4
Fruits	7	3.8
Rice	7	3.8
Bread products	5	2.8
Eggs	5	2.8
Soybean	5	2.8
Chili	4	2.2
Drinks	2	1.1
Total	**181**	**100%**

**Table 4:** Occurrence of each SE gene across all isolates Classical SE genes include *sea*, *seb*, *sec*, *sed* and *see*; Non-classical SE genes include *seg*, *seh*, *sei*, *sej* and *sel*.

Type of SE Genes	Name of SE Genes	%	*n*
**Classical SE genes**	*sea*	18.8	18
	*seb*	7.3	7
	*sec*	5.3	5
	*sed*	0.0	0
	*see*	0.0	0
**Non-classical SE genes**	*seg*	26.0	25
	*sei*	26.0	25
	*seh*	8.3	8
	*sel*	7.3	7
	*sej*	1.0	1
**Total**		100	96

**Table 5:** Percentage of antimicrobial resistance in *S. aureus* isolates from foods and food contact surfaces.

Antimicrobial Class	Antimicrobial Agent Tested in the Study	Percentage of Isolates Showing Resistant Phenotypes (*n*)
**Aminoglycosides**	Amikacin (AK)	0.0%
	Gentamicin (CN)	3.3% (6/181)
**Beta-lactams**	Amoxycillin/Clavulanic Acid (AMC)	0.6% (1/181)
	Ampicillin (AMP)	54.7% (99/181)
	Penicillin G (P)	54.7% (99/181)
**Cephalosporins**	Cefoxitin (FOX)	0.0%
	Ceftriaxone (CRO)	0.0%
**Chloramphenicols**	Chloramphenicol (C)	0.0%
**Fluoroquinolones**	Ciprofloxacin (CIP)	1.7% (3/181)
	Norfloxacin (NOR)	0.6% (1/181)
**Clycopeptides**	Vancomycin (VA)	0.0%
**Macrolides**	Azithromycin (AZM)	8.8% (16/181)
	Rifampicin (RD)	0.0%
**Sulphonamides**	Sulphamethoxazole/Trimethoprim (SXT)	0.0%
**Tetracyclines**	Tetracycline (TE)	14.9% (27/181)

**Table 6:** Percentage of antimicrobial resistance in *S. aureus* isolates classified based on food and food contact surface categories.

Antimicrobial Class	Antimicrobial Agent Tested in the Study	Bread Products (*n* = 5)	Chilli (*n* = 4)	Drinks (*n* = 2)	Eggs (*n* = 5)	Fruits (*n* = 7)	Hand Swabs (*n* = 48)	Meat Dishes (*n* = 17)	Mixed (*n* = 23)	Rice (*n* = 7)	Seafood Dishes (*n* = 26)	Soybean (*n* = 5)	Vegetables (*n* = 32)
**Aminoglycosides**	Amikacin (AK)	0	0	0	0	0	0	0	0	0	0	0	0
	Gentamicin (CN)	0	25	0	0	14	0	0	0	0	4	0	9
**Beta-lactams**	Amoxycillin/ Clavulanic Acid (AMC)	0	0	0	0	0	0	0	0	0	4	0	0
	Ampicillin (AMP)	80	25	50	40	71	60	53	57	57	54	40	47
	Penicillin G (P)	80	25	50	40	71	60	53	57	57	54	40	47
**Cephalosporins**	Cefoxitin (FOX)	0	0	0	0	0	0	0	0	0	0	0	0
	Ceftriaxone (CRO)	0	0	0	0	0	0	0	0	0	0	0	0
**Chloramphenicols**	Chloramphenicol (C)	0	0	0	0	0	0	0	0	0	0	0	0
**Fluoroquinolones**	Ciprofloxacin (CIP)	0	0	0	0	0	2	6	4	0	0	0	0
	Norfloxacin (NOR)	0	0	0	0	0	0	0	4	0	0	0	0
**Clycopeptides**	Vancomycin (VA)	0	0	0	0	0	0	0	0	0	0	0	0
**Macrolides**	Azithromycin (AZM)	0	0	0	20	14	8	0	4	14	12	20	13
	Rifampicin (RD)	0	0	0	0	0	0	0	0	0	0	0	0
**Sulphonamides**	Sulfamethoxazole/ Trimethoprim (SXT)	0	0	0	0	0	0	0	0	0	0	0	0
**Tetracyclines**	Tetracycline (TE)	60	25	0	0	29	15	18	9	0	8	20	19

Legend: 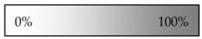.

**Table 7:** Number of classical SE genes detected in *S. aureus* isolates, with the top six *spa* types in 181 isolates.

*Spa* Type	*sea*	*seb*	*sec*
**t5078** (***n* = 73**)	0	0	0
**t084** (***n* = 19**)	0	2	0
**t5521** (***n* = 11**)	11	0	0
**t189** (***n* = 10**)	0	0	0
**t6675** (***n* = 9**)	1	0	0
**t127** (***n* = 6**)	3	1	1

## Data Availability

All data generated or analyzed during this study were included in this article.
